# Pulmonary Consumption

**Published:** 1887-11-26

**Authors:** 


### Turning over New Leaves.

Books for Review should be sent to The Editor, The Lodge, Porchester Square, W.

### PULMONARY CONSUMPTION.

Knowledge, courage, honesty—these are striking characteristics of this book, "Pulmonary Consumption."* Specifics, royal roads, flattering promises of speedy and permanent cure, find no place in its pages. Lies and knavery are common in books that treat of this subject, and they are not exclusively the property of those who are outside the recognised enclosure of physic. Among fully-qualified men there are not wanting wretched compilers who teach what they know to be false, and promise what they are certain they can never fulfil. The public is foolish, superstitious, credulous, and unbelieving at one and the same time. We do not claim for any profession an absolute immunity from error, or a standard of rectitude which reaches to perfection. But we do claim for that profession which is best known to us—the profession of medicine—that its teachers and leaders possess more knowledge of the subject-matter of their business, more practical and experienced skill, and a higher simplicity and honesty of character, than is possible to any uninstructed and unqualified professor of medicine whatsoever. Any unqualified and uninstructed person who professes to know what the most scientific medical men do not know, and to cure where they have failed to cure, stands confessed, by that very fact, a liar and a scoundrel. Among our readers are many unprofessional persons; and we put on record, for their instruction and advantage, this emphatic protest. Reason and homely sense should teach everybody that the blacksmith can make a better horse-shoe than the tailor, and that the sailor can handle a rudder more skilfully than the ploughman. We readily admit that qualified practitioners may be so indolent as to be useless; and that here and there a naturally clever man, though untaught, may effect cures where such practitioners fail. But when we are dealing with the leaders of the medical and surgical professions, there can be no possible comparison between them and any outside pretender whatsoever. Dr. J. B. Williams has spent a lifetime in what may be called the special and technical laboratories devoted to consumption. Following in his father's footsteps, Dr. Theodore Williams has given up the five-and-twenty years of his professional life to the same study. Every possible avenue of knowledge has been open to them both. Neither of them has been under any temptation to study the subject in any other than a purely scientific and practical way. Both of them have been influenced by a laudable anxiety to discover or construct, if possible, some method of treatment which should be effectual in the case of so terrible and widespread a malady. There is every possible inducement for a specialist in phthisis to give himself heart and soul to the study and cure of such a terrible enemy of man. Any man who should discover a really working cure, capable of being easily carried out by the ordinary practitioner, would win for himself a name and reputation second to none in the annals of the benefactors of mankind.

With such unique opportunities as the father and son have possessed, it was to be expected that they would produce a work of high scientific and practical value; and that is exactly what they have done. Like experienced travellers, they have placed before us a record of facts, observations, experiences, and reasonings which, on the subject treated, has no equal in the English language. Alike from the historical, scientific, practical, and philanthropic standpoints, the book is one to be welcomed, and studied with the care and thoroughness due to genuine and original work. Space does not permit us now to enter into details, but the several parts of the book contain matter of the highest interest both to professional and lay readers; and we propose, as opportunity offers, to discuss the various points raised by the authors, especially considering the questions of cause, treatment, and prevention. The subject of phthisis is of the very first importance to professional readers, and especially to students and young practitioners. We do not know where to point them to any such mine of historical and practical wealth as will be found here. We are not saying too much when we express the conviction that it is the duty of every student and young practitioner to make himself master of the contents of the book.

* "Pulmonary Consumption" By J. B. Williams and C. T. Williams. London: Longmans and Co. Second edition, 16s.

